# Single Posthepatic Portosystemic Shunt Communicated With Internal Thoracic Vein and Azygos Continuation of the Caudal Vena Cava in a Dog

**DOI:** 10.1002/vms3.70057

**Published:** 2024-09-24

**Authors:** Ryo Takeuchi, Kumiko Ishigaki, Orie Yoshida, Tatsuya Heishima, Kaito Iida, Kazushi Asano

**Affiliations:** ^1^ Laboratory of Veterinary Surgery, Department of Veterinary Medicine, College of Bioresource Sciences Nihon University Fujisawa Kanagawa Japan

**Keywords:** dog, internal thoracic veins, portosystemic shunt, posthepatic, surgery, umbilical veins

## Abstract

Computed tomography angiography (CTA) was performed under general anaesthesia on a 7‐month‐old toy poodle that was referred with the chief complaints of salivation and neurological symptoms. The CTA revealed a rare form of posthepatic portosystemic shunt (PSS) via the suspected persistent left umbilical vein communicating with the internal thoracic vein in addition to an azygos continuation of the caudal vena cava (CVC). The patient underwent surgery for partial ligation of PSS on Day 4 after the initial examination. On Day 71, after the initial examination, a second surgery was performed for complete ligation. Approximately 10 years have passed since the patient's second surgery, and he is still healthy, and generally in good condition. Although the morphology of the shunt in this case was unusual and was accompanied by an azygos continuation of the CVC, a favourable course of treatment was obtained by ligating the shunt vessel. This case report suggests that CTA can reveal the complex morphological characteristics like our case. Surgical treatment in this case resulted in favourable progress, similar to that in dogs with commonly observed extrahepatic PSS.

## Graphical abstract

Computed tomography angiography revealed a rare form of posthepatic portosystemic shunt (PSS) via the suspected persistent left umbilical vein communicating with the internal thoracic vein in addition to an azygos continuation of the caudal vena cava revealed by computed tomography angiography in a 7‐month‐old, intact male toy poodle. The patient underwent two‐stage surgeries for complete attenuation of posthepatic PSS. Approximately 10 years have passed since the second surgery, and he was still healthy and generally in good condition.

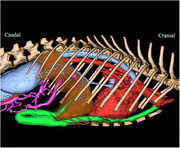

## 1 Introduction

Portosystemic shunts (PSSs) refer to abnormal blood vessels that connect the portal venous system to the systemic circulation and are classified into congenital and acquired types. Congenital PSS (CPSS) occurs in 0.18% of all dogs (Tobias and Rohrbach 2003). Shunt vessels in CPSS can be formed extrahepatically or intrahepatically. Extrahepatic PSS (EHPSS) is more common in small and toy dog breeds (Bossche et al. 2012; Fukushima et al. 2014; Tobias and Rohrback 2003), whereas intrahepatic PSS (IHPSS) occurs mainly in large purebred dogs (Hunt 2004; van Steenbeek et al. 2012).

The EHPSS is thought to be an anastomosis that is caused by an error during embryonic vitelline and cardinal venous system development (Bertolini 2019; Bossche and Steenbeek 2016; Payne, Martin, and Constantinescu 1990). The morphology of EHPSS that is most commonly observed in dogs includes spleno‐caval, left gastro‐phrenic, left gastro‐azygos and right gastro‐caval (Fukushima et al. 2014; Nelson and Nelson 2011; White and Parry 2013, 2015, 2016a, 2016b; White, Shales, and Parry 2017). On the other hand, the underlying mechanisms of IHPSS in the right hepatic division and the central hepatic division have not been clarified: However, one of those in the left hepatic division is thought to be an error in the ductus venosus closure mechanism after birth (Bertolini 2019; Bossche and Steenbeek 2016; Payne, Martin, and Constantinescu 1990; van Steenbeek et al. 2012). A recent study described on the classification of IHPSS as shunt vascular pass between liver lobes in an identifiable fissure (interlobar) or through a specific lobe (intralobar) (Walsh et al. 2023). In the study by Walsh et al., the majority of interlobar IHPSS originate from the left portal branch and are distinguished into four types: patent ductus venosus, left interlobar, right interlobar and ventral interlobar. Meanwhile, the majority of intralobular IHPSS originate from the right portal branch and are distinguished by the right‐lateral lobe or caudate process (Walsh et al. 2023).

In veterinary medicine, posthepatic PSS in which the left umbilical vein is retained and shunts into the internal thoracic vein is extremely rare. A shunt blood vessel was found to course from the left umbilical vein into the falciform fat, passing through the diaphragm, and into the thoracic cavity in one dog and two cats in a previous report (Brockman, Brown, and Holt 1998). However, in these cases, intrathoracic blood vessels were not identified. In addition, few cases in which this rare shunt form of posthepatic PSS and azygos continuation of caudal vena cava (CVC) were diagnosed by computed tomography angiography (CTA), and which were treated surgically, have been reported to date. This case report aimed to describe the clinical characteristics and surgical outcomes of a dog with a rare form of posthepatic PSS, complicated by azygos continuation of the CVC.

## 2 Case Description

The dog was a 7‐month‐old, unneutered male toy poodle, weighing 2.8 kg. Fourteen days before admission, the patient was examined at the referring hospital with chief complaints of salivation and neurological symptoms (e.g. ataxia). A high serum ammonia level (656 µmol/L) was found, leading to suspicion of a PSS. The patient was then referred to our hospital for evaluation and treatment, with medical treatment including lactulose.

**FIGURE 1:**
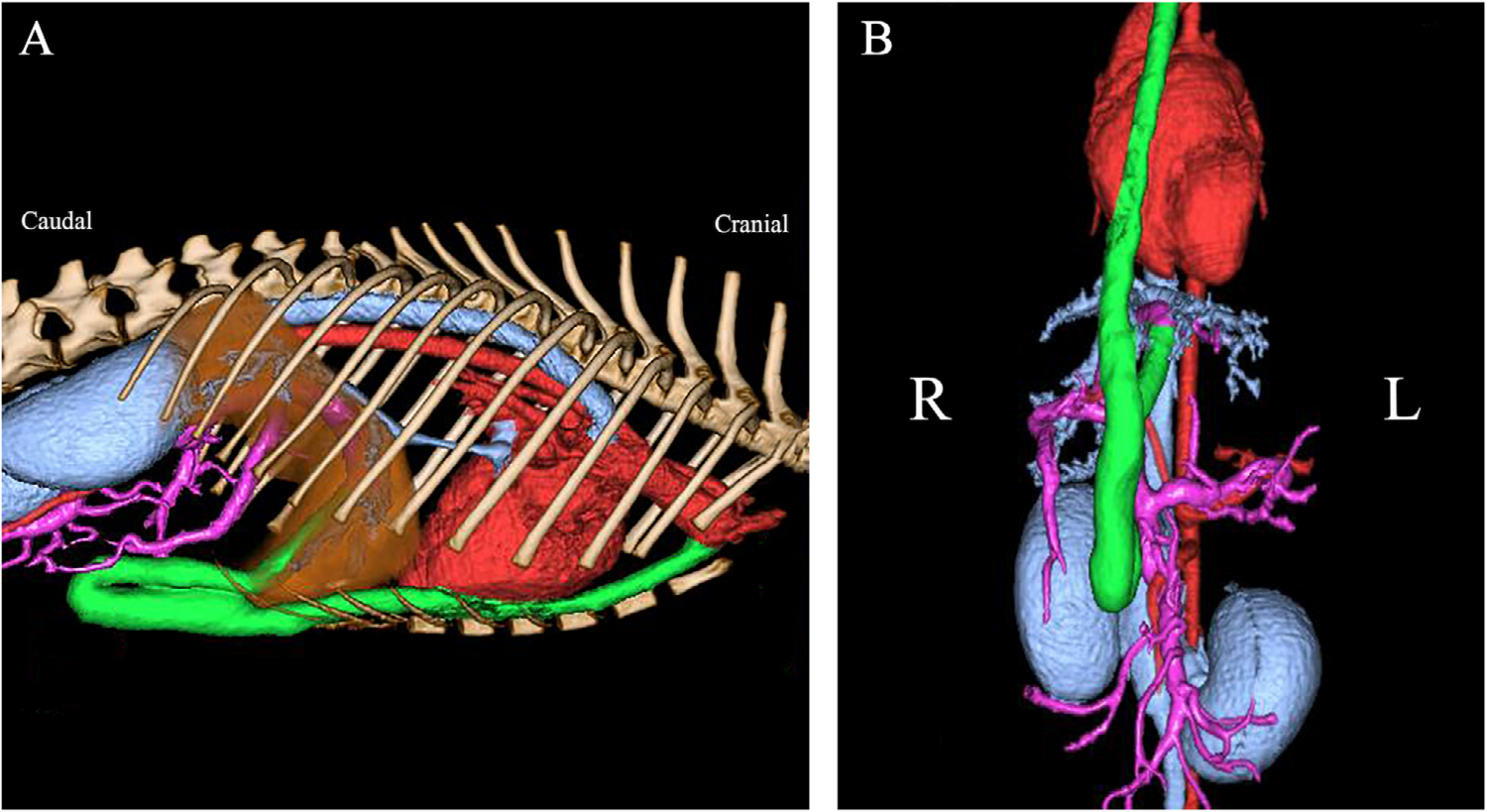
Three‐dimensional computed tomography image obtained before the first surgery in a dog with a rare form of portosystemic shunt with azygous continuation of the caudal vena cava: (A) lateral view; (B) ventral view. An unusual form of extrahepatic portosystemic shunt, in which the suspected left umbilical vein was shunted to the internal thoracic vein, and an azygos continuation of the caudal vena cava, was observed. Vessels are color‐coded for ease of classification: red – artery; blue – vein; pink – portal vein; green – shunt blood vessel.

In the first evaluation, a general physical examination revealed no obvious neurological abnormalities; however, cryptorchidism was detected on both sides. Serum chemistry tests were performed, including total serum protein (3.8 g/dL), albumin (2.0 g/dL), aspartate aminotransferase (78 IU/L), alanine aminotransferase (125 IU/L) and blood urea nitrogen (4 mg/dL). Abnormal values, such as serum total bile acid (TBA) concentration (fasting: 31.5 µmol/L, postprandial: 202.5 µmol/L), were observed (Table 1). A blood coagulation test showed prolonged activated partial thromboplastin time (APTT; 25.6 s) and low anti‐thrombin III (ATIII; 67%). X‐ray examination revealed microhepatica and narrowing of the CVC. Ultrasound examination also revealed a narrowing of the CVC and an abnormal blood vessel extending from the CVC to the azygos vein. Electrocardiography showed normal sinus rhythm and no obvious arrhythmia. CTA was conducted under general anaesthesia and confirmed a posthepatic PSS (suspected left umbilical vein–internal thoracic vein shunt) and azygos continuation of the caudal vein (Figure 1).

**TABLE 1:** Haematology, serum chemistry and coagulation test in a dog with a rare form of portosystemic shunt with azygous continuation of the caudal vena cava.

Parameter	Unit	Initial exam	POD 66^a^	POD 73^b^	Normal range
RBC	10^6^/µL	5.5	5.5	5.5	5.5–8.5
PCV	%	34	37	36	37–55
WBC	/µL	16,600	10,500	12,800	6000–17,000
Stab	/µL	0	105	NA	0–300
Seg	/µL	11,786	2,835	NA	3000–11,500
Lym	/µL	3,154	6,458	NA	1000–4800
Mono	/µL	1,245	525	NA	150–1350
Eos	/µL	415	578	NA	100–750
Plt	10^3^/µL	37	252	377	200–400
TP	g/dL	3.8	5.2	5.6	5.2–8.2
Alb	g/dL	2.0	2.9	2.6	2.7–3.8
Glu	mg/dL	85	89	97	77–125
AST	U/L	78	22	20	0–50
ALT	U/L	125	31	65	10–100
ALP	U/L	228	144	123	23–212
GGT	U/L	4	4	0	0–7
BUN	mg/dL	4	9	7	7–27
Cr	mg/dL	0.3	0.6	0.7	0.5–1.8
T‐CHO	mg/dL	192	351	277	110–320
NH_3_	µmol/L	48	15	0	0–98
Na	mmol/L	148	147	144	134–153
K	mmol/L	5.1	4.1	4.5	3.4–4.6
Cl	mmol/L	119	113	117	105–118
CRP	mg/dL	1.45	0.35	5.6	0–1.00
TBA					
Fasting	µmol/L	31.5	4.1	0.8	
Postprandial	µmol/L	202.5	125.0	44.1	
APTT	s	25.6	13.0	NA	14.0–21.0
PT	s	7.1	6.6	NA	6.0–9.0
Fib	mg/dL	204.7	279.1	NA	200.0–400.0
AT III	%	67	105	NA	> 90

Abbreviations: Alb, albumin; ALP, alkaline phosphatase; ALT, alanine aminotransferase; APTT, activated partial thromboplastin time; AST, aspartate aminotransferase; AT III, antithrombin III; BUN, blood urea nitrogen; Cr, creatinine; CRP, C‐reactive protein; Eos, eosinophil; Fib, fibrinogen; GGT, gamma‐glutamyltransferase; Initial exam, initial examination; Lym, lymphocyte; Mono, monophil; NH_3_, ammonia; PCV, packed cell volume; Plt, platelet count; PT, prothrombin time; RBC, red blood cell count; Seg, segmented neutrophil; Stab, stab neutrophil; TBA, total bile acid; T‐CHO, total cholesterol; TP, total protein; WBC, white blood cell count.

^a^ POD 66, postoperative Day 66 after the first operation, which was before the second operation.

^b^ POD 73, postoperative Day 73 after the first operation, which was 6 days after the second operation.

After receiving sufficient explanation, the owner chose surgical rather than medical treatment for the PSS. The shunt attenuation was performed 4 days after the initial examination. Until surgery, the patient was managed medically with lactulose (Kowa Co. Ltd., Nagoya, Japan) and amoxicillin (Sandoz Co. Ltd., Tokyo, Japan).

The patient was premedicated with 0.04 mg/kg atropine (Mitsubishi Tanabe Pharma Co., Osaka, Japan) injected subcutaneously. Next, 5.9 mg/kg propofol (Mylan Seiyaku Ltd., Tokyo, Japan) was administered intravenously to induce anaesthesia. After endotracheal intubation, mechanical ventilation was performed using isoflurane (IsoFlo; Zoetis, Parsippany‐Troy Hills, NJ, USA) and pure oxygen (2 L/min). For intraoperative and postoperative medical management, 2.5–5.0 µg/kg/min dopamine (Teva Takeda Pharma Ltd., Nagoya, Japan) and 2.5–5.0 µg/kg/min dobutamine (Kyowa Pharmaceutical Industry Co. Ltd., Osaka, Japan) was administered as a continuous infusion. For analgesic management, a continuous infusion of 5.0–30 µg/kg/h remifentanil (Maruishi Pharmaceutical Co., Osaka, Japan) was used during the perioperative period. Cefazolin sodium (Nichi‐Iko Pharmaceutical Co. Ltd., Toyama, Japan) 20 mg/kg was administered intravenously during induction of anaesthesia and every 2 h during general anaesthesia. Preoperative blood coagulation tests revealed abnormalities in APTT and ATIII; thus, whole blood transfusions (total volume of 100 mL) were administered during and post‐surgery to improve haemostasis.

**FIGURE 2:**
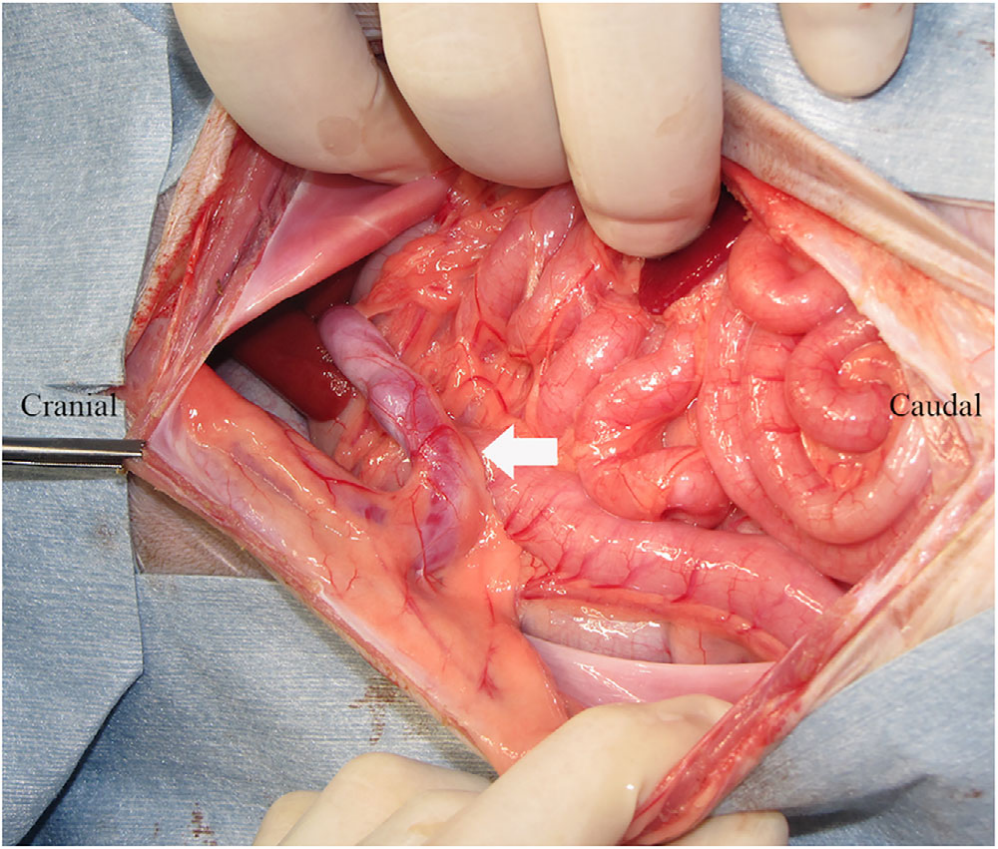
Photograph of intraperitoneal exploration during the first surgery in a dog with a rare form of portosystemic shunt with azygous continuation of the caudal vena cava. The intraperitoneal gastrointestinal tract was retracted caudally, and the shunt blood vessel (white arrow) in which the suspected left umbilical vein ran into the falciform fat could be observed.

**FIGURE 3:**
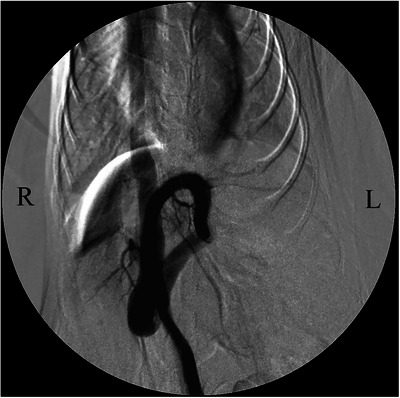
Intraoperative mesenteric portovenogram in dorsoventral projection before temporary closure of the shunt vessel using atraumatic bulldog forceps during the first surgery in a dog with a rare form of portosystemic shunt with azygous continuation of the caudal vena cava. Blood flow to the liver was poor. A shunt blood vessel (suspected left umbilical vein‐internal thoracic vein shunt) from the abdominal cavity to the thoracic cavity was visualized.

**FIGURE 4:**
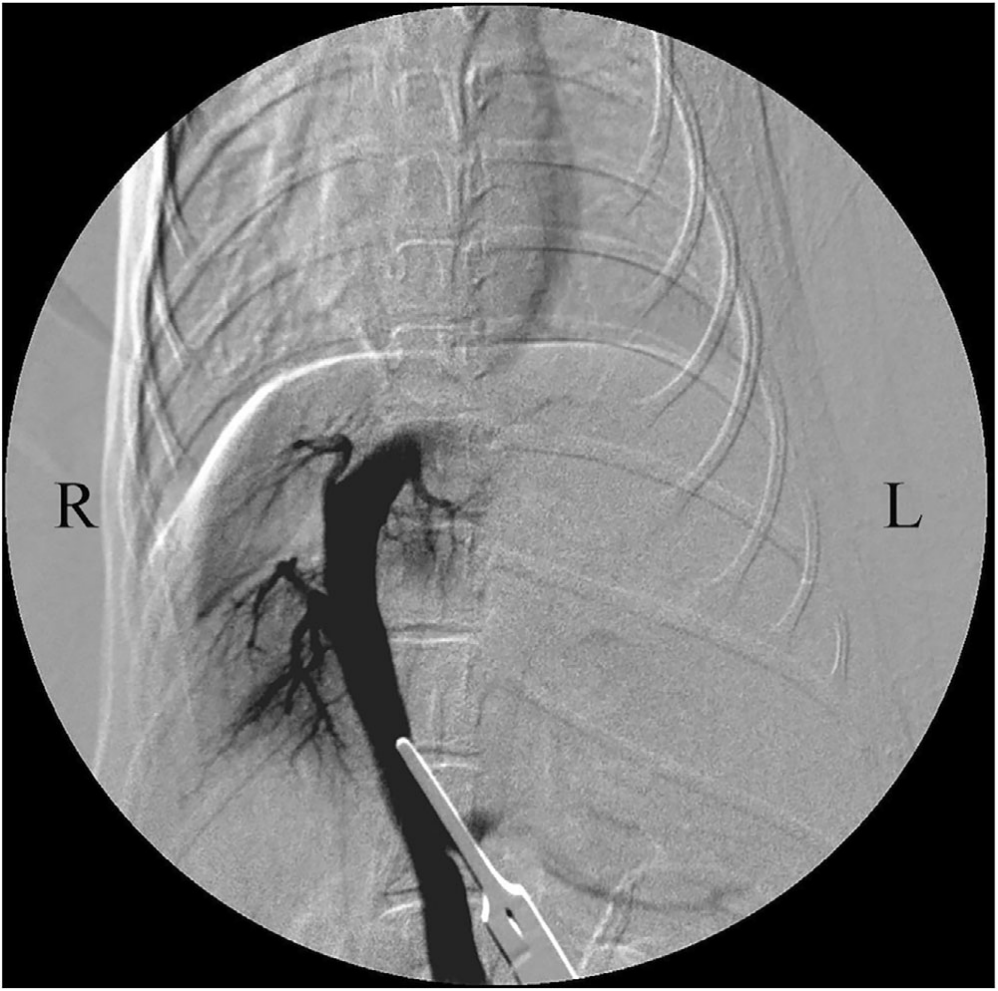
Intraoperative mesenteric portovenogram in dorsoventral projection after temporary closure of the shunt vessel using atraumatic bulldog forceps during the first surgery in a dog with a rare form of portosystemic shunt with azygous continuation of the caudal vena cava. The portal vein blood flow toward the cranial side of the closure site was confirmed to be poor, and the intrahepatic portal vein branches were clearly not developed. No abnormal blood vessels other than shunt blood vessels were observed.

The patient was placed in dorsal recumbency and prepared for abdominal surgery under general anaesthesia. An abdominal midline incision was made, a puncture incision was made in the abdominal wall slightly to the left of the linea alba and the abdomen was opened along the linea alba. The stomach and intestinal tract were retracted caudally, the diaphragm was incised, thoracotomy was performed and it was visually confirmed that the shunt blood vessel (left umbilical vein–internal thoracic vein shunt) ran from the abdominal cavity to the thoracic cavity (Figure 2). In addition, a distended azygos vein was confirmed in the abdominal cavity. One layer of the omentum was manually torn, the stomach was lifted and the shunt blood vessels were separated from the surrounding tissue and exposed. A cannula was placed in the mesenteric vein using a 24‐G indwelling needle (Surflow indwelling needle, Terumo Co. Ltd., Tokyo, Japan), and portal venous pressure was measured as 3 mmHg. Intraoperative mesenteric portovenography (IOMP) was performed by administering iohexol (350 mg I/mL) through the mesenteric vein. Nearly no blood flow to the liver was observed, whereas shunt blood flow from the left umbilical vein to the internal thoracic vein was noted (Figure 3). IOMP was performed by temporarily closing the shunt vessel using atraumatic bulldog forceps. Consequently, portal venous blood flow towards the cranial side of the closure site was poor. No abnormal blood flow was observed except for the shunt vessels, and portal venous pressure rose to 11 mmHg (Figure 4). When the shunt blood vessel was temporarily blocked, cyanosis was observed in the intestinal tract and pancreas and hyperperistalsis was seen in the gastrointestinal tract. Therefore, partial ligation was performed using a coated braided nylon suture, size 0 (Surgilon; Medtronic Inc., Minneapolis, MN, USA) to achieve a final portal pressure of 6 mmHg. A portion of the left hepatic lateral lobe margin was obtained for histopathological examination. Thereafter, the patient was castrated to address the cryptorchidism, and the abdomen was closed in a conventional manner. Histopathological examination of the biopsied liver revealed poor vascular development in the portal system, irregular lobular structure and mild atrophy of hepatocytes.

The operative time was 144 min. The patient recovered well after anaesthesia. No abnormalities were observed in the patient's general condition during hospitalization. The patient was prescribed a liver therapy diet. Cefalexin (FUJIFILM Toyama Chemical Co. Ltd., Tokyo, Japan) and lactulose (3 mL/head twice daily) (Kowa Co. Ltd.) were administered orally. The patient was discharged from the hospital on postoperative Day 8 and was prescribed cefalexin (FUJIFILM Toyama Chemical Co. Ltd.) for 1 week after discharge. Stitches were removed 2 weeks post‐surgery at the referring hospital.

**FIGURE 5:**
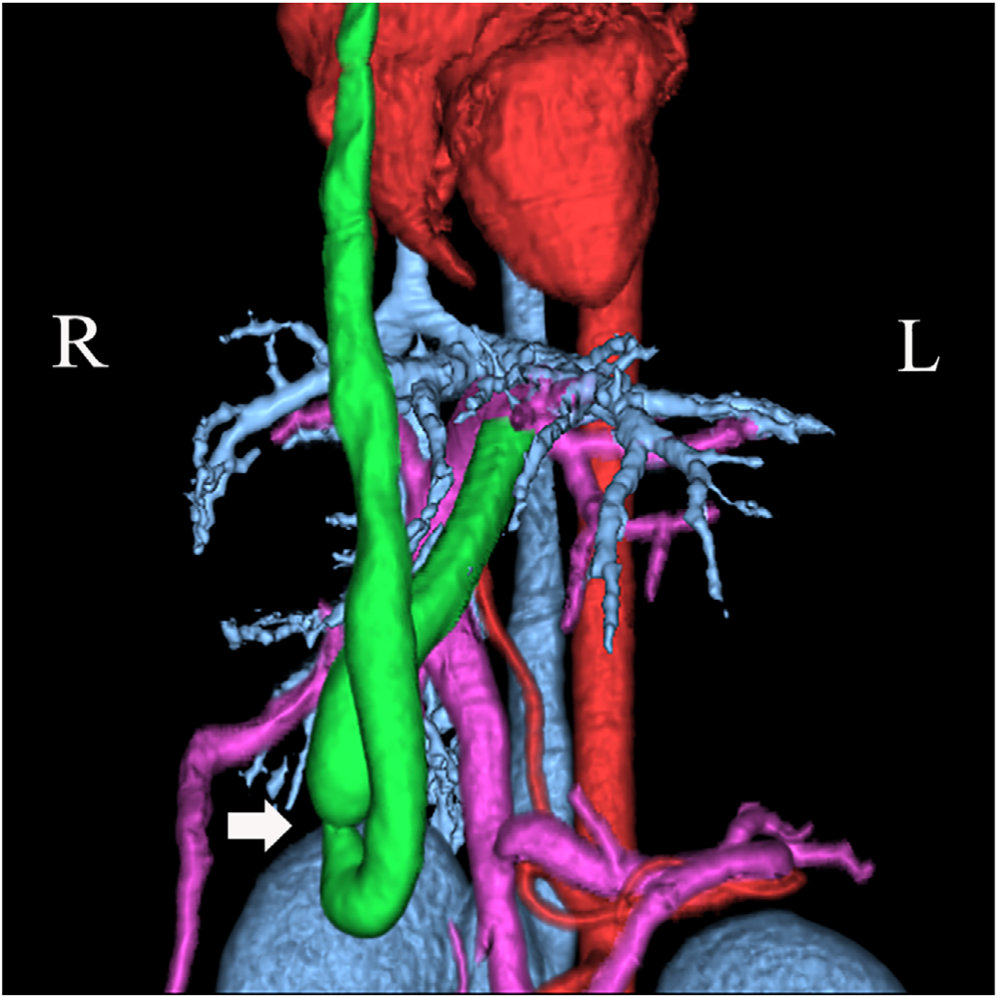
Three‐dimensional computed tomography image obtained before the second surgery in a dog with a rare form of portosystemic shunt with azygous continuation of the caudal vena cava. Compared to the first surgery, we observed narrowing of the shunt vessel (white arrow) and development of intrahepatic portal vein branches due to partial ligation. Vessels are color‐coded for ease of classification: red – artery; blue – vein; pink – portal vein; green – shunt blood vessel.

**FIGURE 6:**
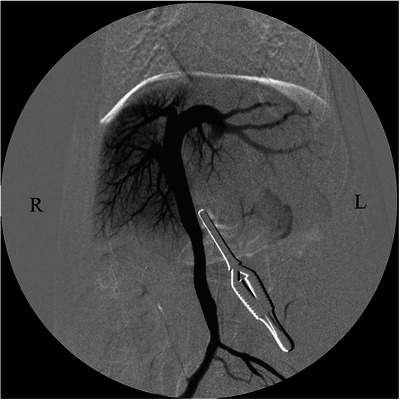
Intraoperative mesenteric portovenogram in dorsoventral projection after the shunt vessel was temporarily closed using atraumatic bulldog forceps during the second surgery in a dog with a rare form of portosystemic shunt with azygous continuation of the caudal vena cava. The intrahepatic portal vein branches were visualized up to the tertiary branch. Development of the intrahepatic portal vein was confirmed as compared with that at the time of the first surgery.

On Day 66 after the first surgery, blood examinations revealed that the abnormal values observed at the first visit had improved (Table 1). A second surgery was performed to completely occlude the shunt vessel 67 days after the first surgery. Anaesthesia was administered as in the first surgery, but no blood transfusion was performed. When CTA was performed under general anaesthesia before surgery, narrowing of the shunt blood vessel and development of intrahepatic portal vein branches were observed (Figure 5). Laparotomy was performed using the same technique as in the first surgery, and the portal vein pressure was measured at 2 mmHg, which was 3 mmHg after temporary occlusion. Here, no cyanosis in the pancreas or intestines or hyperperistalsis in the gastrointestinal tract was observed. When IOMP was performed, the development of intrahepatic portal vein branches was confirmed (Figure 6). The shunt vessel was completely ligated using a coated braided nylon suture, size 0 (Surgilon), and portal vein pressure after complete ligation was 4 mmHg. Finally, a biopsy of the left hepatic lateral lobe margin was also performed. The abdominal wall, subcutaneous tissue and skin were routinely closed. Histopathological examination of the biopsied liver showed significant improvement in vascular structure and portal vein diameter.

The operative time of the second surgery was 43 min. During the second hospitalization, the patient's general condition remained stable, and he was discharged on postoperative Day 6 after continuing medical management with cefovecin sodium (Convenia; Zoetis) and dietary management. No abnormal values were detected in blood tests at the time of discharge, and TBA was relatively low (fasting: 0.8 µmol/L, postprandial: 44.1 µmol/L) (Table 1). Therefore, oral medication and dietary management were not prescribed at discharge. The stitches were removed at the referring hospital. Approximately 10 years have passed since the second surgery, and the patient has progressed well, without any medical treatments.

## 3 Discussion

During development, the venous system that returns blood to the heart differentiates in parallel with the arterial system (Hyttel 2009). The vitelline veins run from the yolk sac to the sinus venosus of the developing heart, and the umbilical vein bypasses the developing liver on its way to the heart. During development, the right umbilical vein regresses, whereas the left umbilical vein forms the portal sinus as a connection with the right vitelline vein (Hikspoors et al. 2017). Additionally, as a connection to the right hepatocardiac channel, the left umbilical vein forms a ductus venosus with the proximal portion of the right vitelline vein (Hikspoors et al. 2017; Hyttel 2009). The distal part of the right vitelline vein develops into the portal vein (Hyttel 2009). The ductus venosus is thought to close within 3–6 days after delivery due to cessation of blood flow in the umbilical vein and a decrease in pressure within the portal sinus (Lohse and Suter 1977; Meyer and Lind 1966; Oliveira et al. 1979). In this case, although the ductus venosus had closed, the left umbilical vein remained, which may have induced vascular malformation during embryonic development. In medicine, in healthy subjects, the internal thoracic vein and the vein within the falciform ligament anastomose. Furthermore, the vein within the falciform ligament and the left branch of the intrahepatic portal vein anastomose in patients with portal hypertension (PH) (Ibukuro et al. 2008). In veterinary medicine, when the cranial vena cava is occluded, the main collateral channels are the internal thoracic veins (Ricciardi and Casali 2020). The internal thoracic vein may be used as a collateral route if normal blood flow is obstructed. Shunt formation of the portal vein to the left internal thoracic vein in dogs with an acquired PSS accompanied by PH has also been reported (Ricciardi 2016). Blood does not flow easily to the liver due to primary hypoplasia of the portal vein, and the blood flow vector in the remaining left umbilical vein changes, resulting in the use of the internal thoracic vein. Thus, shunt blood vessels may have been acquired. However, on the basis of the clinical course and the results of histopathological examination of the liver, an acquired PSS was unlikely in this case. The condition of this patient differed from IHPSS associated with patent ductus venosus, and we consider that a complex error during development resulted in an unusual form of posthepatic PSS.

A previous study advocated to classify the shunt vessel as ‘intrahepatic’ or ‘extrahepatic’, depending on whether they originated cranial or caudal to the gastroduodenal vein (Walsh et al. 2023). The previous study showed the case that the ventral interlobar IHPSS arose from the left portal branch and extended ventral to the porta hepatis, running cranially and then looping caudoventrally passing through the fissure for round ligament (caudal part of the central longitudinal fissure). This form of IHPSS is described as a vessel branching into the branch of the liver parenchyma, passing between the left medial and quadrate lobes, and anastomosing with the paraumbilical vein at the falciform ligament. In medicine, the paraumbilical vein is known to terminate in the parenchyma of the quadrate and left lateral lobes of the liver, with several branches communicating with peripheral portal branches (Lin, Lunderquist, and Hägerstrand 1984). The origin of the shunt vessel in our case was located cranial to the gastroduodenal vein, and the shunt vessel passed between the left medial and quadrate lobes: However, the shunt vessel directly originated from the main left portal branch. In addition, the shunt vessel was running away from the liver and folded back at the umbilicus outside the liver parenchyma. The left umbilical vein travels from the umbilicus to the left portal branch and eventually becomes the round ligament of the liver (ligamentum teres). At the time of the first surgery in this patient, we searched for but could not find the round ligament. Therefore, the shunt vessel was concluded to potentially originate from a persistent left umbilical vein. We first considered that this case was categorized in a posthepatic EHPSS due to the shunt vessel running out of the liver parenchyma. However, according to the previous study (Walsh et al. 2023), this case may be categorized as IHPSS, and its classification is controversial. In addition, the origin of the shunt vessel in our case could not be definitively identified as the persistent left umbilical vein. Therefore, the shunt vessel in our case was concluded to be posthepatic PSS communicated with the internal thoracic vein.

The incidence of abnormal development of the CVC in normal dogs is approximately 1% (Buchanan 1992). The CVC and the azygos vein form due to the appearance, anastomosis and disappearance of three veins, such as the caudal cardinal vein, the subcardinal vein and the supra‐cardinal vein, during embryonic development (Hyttel 2009). Azygos continuation of the CVC is a malformation that occurs when the right subcardinal vein fails to connect with the right vitelline vein and switches the blood route to the right supra‐cardinal vein. Consequently, blood flow in the caudal region of the CVC flows into the heart through the azygos vein and cranial vena cava (Fischetti and Kovak 2008). In the present case, EHPSS and azygos continuation of the CVC co‐occurred. A similar co‐occurrence has been reported in previous literature (Fischetti and Kovak 2008; Hunt et al. 1998; Zwingenberger, Spriet, and Hunt 2011). Although the details of the relationship among these vascular malformations are unknown, it may be due to an anastomotic error in the right vitelline vein during development.

CPSS often presents with clinical signs at a young age (Fukushima et al. 2014; Tobias and Rohrbach 2003). Symptoms are diverse and commonly include poor growth, weight loss, anorexia, vomiting and diarrhoea. Additionally, neurological symptoms caused by hepatic encephalopathy appear intermittently and have been reported to include ataxia, weakness, depression and seizures (Center and Magne 1990; Mehl et al. 2005; Radlinsky and Fossum 2018). Although this case was accompanied by azygos continuation of the CVC, this congenital malformation is generally said to be asymptomatic (Fischetti and Kovak 2008; Lockwood et al. 2018). In patients with CPSS, commonly observed serum chemistry abnormalities include decreases in albumin, total serum protein and urea nitrogen. Decreased cholesterol and blood sugar levels and increased liver enzyme levels have also been reported (Center and Magne 1990). In addition, serum ammonia concentration and serum TBA concentration are used as indicators of decreased liver function, and individuals with CPSS often show abnormal values. A previous report with a similar shunt morphology to this case showed clinicopathological features similar to EHPSS, which is a common shunt morphology (Brockman, Brown, and Holt 1998). The age of onset, clinical signs and blood test results in this case were similar to those previously reported in dogs with EHPSS. On the basis of these facts, this diagnostic method can be used for EHPSS in general and even rare forms of posthepatic PSS such as this case.

In recent years, diagnostic techniques have improved, and many reports have anatomically explained the shunt morphology of EHPSS by using CTA, IOMP and intraoperative macroscopic findings (Fukushima et al. 2014; Nelson and Nelson 2011; White and Parry 2013, 2015, 2016a, 2016b; White, Shales, and Parry 2017, White, Parry, and Shales 2018, White et al. 2020). A previous report concluded that, in normal dogs, CTA shows more detail of the extrahepatic portal vein and its branches compared with IOMP (Parry and White 2015). Additionally, CTA is reportedly able to visualize downstream branches of the portal vein that cannot be seen with IOMP (Parry and White 2015; White and Parry 2013, 2016a, 2016b). However, according to a study comparing image findings from preoperative CTA and IOMP, IOMP images obtained by temporarily fully ligation (TFL) of the shunt vessel (TFL‐IOMP) provided important findings during surgery (Parry and White 2018). Other specific benefits of implementing TFL‐IOMP include the ability to measure portal venous pressure, visualize intrahepatic portal vein branches, confirm the presence of only one shunt vessel and confirm whether the shunt vessel is closed at the appropriate site (Lee et al. 2006; Lipscomb et al. 2009; Parry and White 2017; White, Macdonald, and Burton 2003). Therefore, using CTA and IOMP together makes it possible to identify the anatomical characteristics of the shunt blood vessel in more detail and obtain information that is useful as surgical indicators. Even in posthepatic PSS with a rare shunt form, such as that in this case, we were able to detect the anatomical characteristics of the shunt vessel using CTA and IOMP. CTA is an effective preoperative diagnostic tool and should be performed to determine surgical plans and treatment strategies.

The shunt form of posthepatic PSS in our case was extremely rare, and its anatomical characteristics have not been reported in detail in the veterinary literature. In this case, the shunt blood vessel running from the left umbilical vein into the falciform fat was confirmed under abdominal surgery, and the site for surgical occlusion was successfully exposed. However, we considered that small shunt vessels, which were not visualized by CTA or IOMP, may exist. Therefore, during the first surgery, a diaphragmatic incision was made and the chest was opened, and the intrathoracic course of the shunt blood vessel from the left umbilical vein to the internal thoracic vein was visually confirmed. In future, when performing surgical treatment on posthepatic PSS patients with this type of shunt, thoracotomy may not be required, as laparotomy alone will be sufficient.

Surgical treatment is considered an important treatment to close the shunt vessel in dogs and cats with PSS (Radlinsky and Fossum 2018). However, artificially closing the shunt blood vessel may lead to PH. Complete ligation was possible in 12%–52% of cases in dogs with EHPSS, and the perioperative mortality rate because of ligation was 0%–29% (Faverzani et al. 2003; Hottinger, Walshaw, and Hauptman 1995; Hurn and Edwards 2003; Hunt and Hughes 1999; Kummeling et al. 2006, Kummeling et al. 2010; Murphy et al. 2001; Smith et al. 1995; Szatmári et al. 2004; Winkler et al. 2003). When complete ligation is not possible, various techniques have been reported, including partial ligation, ameroid constrictor placement and cellophane banding (Radlinsky and Fossum 2018; Serrano et al. 2019; White, Parry, and Shales 2018), but the postoperative results are similar (Serrano et al. 2019). When performing surgical ligation, measuring portal vein pressure intraoperatively is crucial. As a guideline for safe ligation, portal venous pressure should be kept below 12–18 mmHg, and the fluctuation range of portal venous pressure after ligation should not exceed 7 mmHg, as compared to the portal venous pressure before ligation (Breznock et al. 1983; Johnson, Armstrong, and Hauptman 1987). A report that measured the shunting rate of PSS using radioisotopes (Daniel et al. 1991) found that 60%–94% of portal blood bypassed the hepatic circulatory system, resulting in liver failure. In the first surgery in this case, the portal vein pressure was not high when the shunt vessel was temporarily completely blocked. However, when the shunt fraction was calculated based on a previous report (Washizu et al. 2004), it was as high as 73%. Considering the intraoperative findings, partial ligation rather than complete ligation was appropriate. A previous report of a dog with a shunt configuration similar to the present case did not provide details regarding postoperative follow‐up. However, 2 years after the ligation procedure, the animal visited a veterinary hospital with a chief complaint other than PSS (cervical spinal discomfort), and the blood test results at that time were normal (Brockman, Brown, and Holt 1998). Although ligation was performed twice in the present case, the patient's progress was good, and he is still alive. These results suggest that surgical treatment may yield a good prognosis for rare forms of posthepatic PSS, such as the present case.

In conclusion, although this case involved an unusual form of posthepatic PSS in which the suspected persistent left umbilical vein shunted to the internal thoracic vein, the clinical features and surgical outcomes were similar to those of common EHPSSs. This case report indicated that CTA is a very effective tool for diagnosis and could anatomically identify rare forms of shunt vessels like our case.

## Author Contributions

**Ryo Takeuchi**: conceptualization (supporting), data curation (supporting), investigation (supporting), writing – original draft preparation (equal), writing – review and editing (equal). **Kumiko Ishigaki**: conceptualization (supporting), data curation (supporting), investigation (supporting), methodology (supporting), project administration (supporting), supervision (supporting), visualization (supporting), writing – original draft preparation (equal), writing – review and editing (equal). **Orie Yoshida**: conceptualization (supporting), data curation (supporting), investigation (supporting), methodology (supporting), writing – original draft preparation (equal), writing – review and editing (equal). **Tatsuya Heishima**: conceptualization (supporting), data curation (supporting), investigation (supporting), methodology (supporting), visualization (supporting), writing – original draft preparation (equal), writing – review and editing (equal). **Kaito Iida**: conceptualization (supporting), data curation (supporting), investigation (supporting), methodology (supporting), visualization (supporting), writing – original draft preparation (equal), writing – review and editing (equal). **Kazushi Asano**: conceptualization (lead), data curation (lead), investigation (lead), methodology (lead), project administration (lead), supervision (lead), visualization (supporting), writing – original draft preparation (equal), writing – review and editing (equal).

## Ethics Statement

All procedures were approved by the Ethical Committee of Nihon University Animal Medical Center.

## Consent

Informed owner consent was obtained from the patient's owner prior to the first evaluation.

## Conflicts of Interest

The authors declare no conflicts of interest.

### Peer Review

The peer review history for this article is available at https://publons.com/publon/10.1002/vms3.70057.

## Data Availability

All data supporting the conclusions of this article are included within the article.
